# Indirect effects of 13-valent pneumococcal conjugate vaccine on pneumococcal carriage in children hospitalised with acute respiratory infection despite heterogeneous vaccine coverage: an observational study in Lao People’s Democratic Republic

**DOI:** 10.1136/bmjgh-2021-005187

**Published:** 2021-06-09

**Authors:** Jocelyn Chan, Jana Y R Lai, Cattram D Nguyen, Keoudomphone Vilivong, Eileen M Dunne, Audrey Dubot-Pérès, Kimberley Fox, Jason Hinds, Kerryn A Moore, Monica L Nation, Casey L Pell, Anonh Xeuatvongsa, Manivanh Vongsouvath, Paul N Newton, Kim Mulholland, Catherine Satzke, David A B Dance, Fiona M Russell, the PneuCAPTIVE Lao PDR Research Group, Toukta Bhounkhoun

**Affiliations:** 1 Infection and Immunity, Murdoch Childrens Research Institute (MCRI), Parkville, Victoria, Australia; 2 Department of Paediatrics, The University of Melbourne, Melbourne, Victoria, Australia; 3 Microbiology Laboratory, Mahosot Hospital, Lao-Oxford-Mahosot Hospital-Wellcome Trust Research Unit (LOMHWRU), Vientiane, Vientiane, Lao People's Democratic Republic; 4 Unité des Virus Émergents, UVE: Aix-Marseille Univ - IRD 190 - Inserm 1207 - IHU Méditerranée Infection, Marseille, France; 5 Regional Office for the Western Pacific, World Health Organization (WHO), Manila, Philippines; 6 Institute for Infection and Immunity, St George’s University of London, London, UK; 7 BUGS Bioscience London Bioscience Innovation Centre, London, UK; 8 National Immunization Programme, Ministry of Health, Vientiane, Lao People's Democratic Republic; 9 Mahosot Hospital, Vientiane, Lao People's Democratic Republic; 10 Centre for Tropical Medicine and Global Health, University of Oxford, Oxford, Oxfordshire, UK; 11 Faculty of Infectious and Tropical Diseases, London School of Hygiene & Tropical Medicine, London, London, UK; 12 Department of Microbiology and Immunology, The University of Melbourne at the Peter Doherty Institute for Infection and Immunity, Melbourne, Victoria, Australia; 13 Centre for International Child Health, Department of Paediatrics, The University of Melbourne, The Royal Children's Hospital, Parkville, Victoria, Australia

**Keywords:** vaccines, epidemiology

## Abstract

**Introduction:**

Empiric data on indirect (herd) effects of pneumococcal conjugate vaccines (PCVs) in settings with low or heterogeneous PCV coverage are limited. The indirect effects of PCV, which benefits both vaccinated and non-vaccinated individuals, are mediated by reductions in vaccine-type (VT) carriage (a prerequisite for disease). The aim of this study among hospitalised children in Lao People’s Democratic Republic (Lao PDR) is to determine the effectiveness of a 13-valent PCV (PCV13) against VT pneumococcal nasopharyngeal carriage (direct effects) and the association between village-level PCV13 coverage and VT carriage (indirect effects).

**Methods:**

Pneumococcal nasopharyngeal carriage surveillance commenced in December 2013, shortly after PCV13 introduction (October 2013). We recruited and swabbed children aged 2–59 months admitted to hospital with acute respiratory infection. Pneumococci were detected using *lytA* quantitative real-time PCR and serotyped using microarray. PCV13 status and village-level PCV13 coverage were determined using written immunisation records. Associations between both PCV13 status and village-level PCV13 coverage and VT carriage were calculated using generalised estimating equations, controlling for potential confounders.

**Results:**

We enrolled 1423 participants and determined PCV13 coverage for 368 villages (269 863 children aged under 5 years). By 2017, median village-level vaccine coverage reached 37.5%, however, the IQR indicated wide variation among villages (24.1–56.4). Both receipt of PCV13 and the level of PCV13 coverage were independently associated with a reduced odds of VT carriage: adjusted PCV13 effectiveness was 38.1% (95% CI 4.1% to 60.0%; p=0.032); and for each per cent increase in PCV13 coverage, the estimated odds of VT carriage decreased by 1.1% (95% CI 0.0% to 2.2%; p=0.056). After adjustment, VT carriage decreased from 20.0% to 12.8% as PCV13 coverage increased from zero to 60% among under 5.

**Conclusions:**

Despite marked heterogeneity in PCV13 coverage, we found evidence of indirect effects in Lao PDR. Individual vaccination with PCV13 was effective against VT carriage.

**Key questions**

**What is already known?**

- There is ample evidence from high-income countries that pneumococcal conjugate vaccines (PCVs) reduce the burden of pneumococcal disease in vaccinated and unvaccinated populations through both direct and indirect (herd) effects. The indirect effects comprise a substantial component of overall vaccine impact, contributing to the cost-effectiveness of the vaccine. This evidence is largely based on data from invasive pneumococcal disease surveillance, which can be challenging to implement in resource limited settings that do not routinely collect blood or cerebrospinal fluid samples as part of clinical care, since surveillance requires large numbers of samples to detect a rare outcome.
- Pneumococcal carriage surveillance provides an alternative method that is well suited to monitoring indirect effects, since carriage is a prerequisite for disease and the indirect effects of PCV are mediated by reductions in vaccine-type (VT) carriage and transmission.

**What are the new findings?**

- Our adjusted model predicts that in Lao People’s Democratic Republic (Lao PDR), the prevalence of VT carriage decreased 36%, from 20% to 12.8%, through increases in PCV13 coverage alone (from 0% to 60% among children under 5 years old). Individual vaccination further protects vaccinated individuals against VT carriage by 39.1%.
- Our findings challenge preconceptions that high PCV coverage is required for substantial indirect protection and suggests that substantial benefits can be observed with lower and heterogeneous PCV coverage.

**What do the new findings imply?**

- Using an innovative approach, we were able to generate robust estimates for both the direct and indirect effects of PCV13 against VT carriage in Lao PDR. This is one of the few studies using pneumococcal carriage among hospitalised children with acute respiratory infection to evaluate PCV impact. Sampling children admitted to hospital allows countries to establish surveillance quickly in an accessible population.
- Our results have important implications for the applicability of reduced dose schedules in low coverage settings. Reduced dose schedules, comprising two rather than three or four doses, have the potential to substantially reduce PCV programme costs but rely on indirect effects to maintain vaccine impacts. Therefore, understanding the determinants of indirect effects in a range of settings is global research priority.

## Background

Pneumococcal disease is a leading cause of morbidity and mortality in children, with most cases occurring in low-income and middle-income countries (LMICs).^1^ The introduction of pneumococcal conjugate vaccines (PCVs) has reduced pneumococcal disease in many settings.^2 3^ The vaccines’ benefits have also extended to unvaccinated groups through indirect effects.^4^ This is achieved by the reduction of vaccine-type (VT) pneumococcal carriage among vaccinated children which interrupts transmission of pneumococci to vaccinated and non-vaccinated contacts.^5^

Understanding indirect effects is vital as the cost-effectiveness of the vaccine greatly improves when the protection afforded to the unvaccinated population is considered.^6 7^ Furthermore, the potential role for a reduced-dose PCV schedule, once VTs are under control, is of great interest globally.^8^ Reduced dose schedules use two doses (eg, 1+1) rather than the standard three and therefore substantially reduce programme costs. However, the success of a 1+1 schedule relies on sustained indirect effects to protect children who have only received one primary dose in infancy and therefore likely to have suboptimal direct protection during the time they are most at risk of disease.^8^ Therefore, being able to measure and monitor indirect effects in different settings is critical.

Measurement of PCV impact on disease is challenging and resource-intensive.^9^ Many LMICs are unable to conduct robust pneumococcal disease surveillance to monitor indirect effects, making it very difficult to evaluate the full benefit of PCV in their population.^9^ An alternative approach for assessing PCV impact is using surveillance of nasopharyngeal (NP) pneumococcal carriage.^10^ Pneumococcal carriage is common and generally asymptomatic.^5^ It is the primary means of pneumococcal transmission and a prerequisite for invasive disease.^5^ PCVs reduce carriage of VTs in the nasopharynx among vaccinated individuals, thereby reducing transmission and providing indirect effects to both vaccinated and non-vaccinated individuals.^10^

It is often assumed that high PCV coverage is required to interrupt VT pneumococcal transmission and achieve substantial indirect effects (or indeed the elimination of VT pneumococcal disease), since near-elimination has predominantly been demonstrated in countries with greater than 90% vaccine coverage.^8^ However, two observational studies from the USA suggest that statistically significant indirect effects against pneumococcal VT carriage can be achieved at 58%–75% coverage among children under 5 years of age,^11 12^ but evidence from LMICs are lacking.^4^

Measuring indirect effects is challenging. Since indirect effects benefit both vaccinated and non-vaccinated people within a population, it is typically measured through comparisons pre-PCV and post-PCV introduction. However, these studies need to take into account extraneous factors which affect patterns of pneumococcal carriage and disease over time, such as changes in smoking rates or prevalence of household crowding.^13^ Furthermore, baseline data for comparisons pre-PCV and post-PCV may not always be available.^10^ In recognition of these limitations, a study from Bangladesh used a novel approach to assess the indirect effects of cholera vaccine which compared the rates of cholera in communities with varying levels of vaccine coverage.^14^ For this study, we adapted these methods to evaluate the direct and indirect effects of PCV.^15^

In October 2013, Lao People’s Democratic Republic (Lao PDR) became one of the first countries in South-East Asia to introduce PCV13 into their Expanded Programme of Immunisation programme using a 3+0 schedule, supported by Gavi, the Vaccine Alliance. Lao PDR has a high child mortality rate,^16^ and pneumonia is estimated to account for 20% of both deaths and hospitalisations in children 1–59 months of age.^17 18^ From cross-sectional carriage surveys pre-PCV and 2 years post-PCV in Lao PDR, there was some evidence of indirect effects following PCV13 introduction, with a reduction in the carriage of PCV13 serotypes (adjusted prevalence ratio (aPR) 0.74 (95% CI 0.43 to1.27)) among infants too young to be vaccinated, but the confidence intervals included the null value (ie, an aPR of 1.00).^19^

For this study, we hypothesised that an inverse association exists between village-level PCV13 vaccination coverage in children under 5 years old (the age group most responsible for transmission) and the risk of VT pneumococcal carriage among both vaccinated and undervaccinated children.^20^ To investigate this, we established pneumococcal NP carriage surveillance among children with acute respiratory infection (ARI), commencing alongside PCV13 introduction in Lao PDR. Therefore, in children hospitalised with ARI we aimed to: (1) describe trends in VT carriage over time following PCV13 introduction; (2) investigate the association between PCV13 coverage at each child’s village of residence with the VT carriage (ie, indirect effects) and (3) calculate the adjusted PCV13 vaccine effectiveness (VE) in the same population (ie, direct effects).

## Methods

### Study setting

This study was conducted in Vientiane, the capital of Lao PDR, where 12.7% of the population resides.^21^ Lao PDR is divided into three administrative tiers: province, district and village. Participants were recruited at Mahosot Hospital, a tertiary referral central hospital located within Vientiane Province, which has nine districts, 485 villages, and an average population of 1693 people per village, according to the 2015 census.^22^ PCV13 was introduced into the national immunisation programme in October 2013 using a 3+0 schedule, administered at 6, 10 and 14 weeks of age.^23^ During the initial PCV13 roll-out, there was a catch-up programme, comprised of a single dose, for infants up to 12 months of age. Immunisations are administered at designated village health centres or district hospitals and recorded in both hand-written registers and the parent-held mother child health (MCH) cards. Vaccination coverage data are collated and reported to the Ministry of Health for routine monitoring of immunisation coverage.

### Study design

In this observational study, we enrolled children 2–59 months of age admitted with ARI and obtained an NP swab to detect pneumococcal carriage. This analysis was part of a multi-site study across the Asia-Pacific region examining the relationship between PCV13 coverage and indirect effects in Lao PDR, Mongolia and Papua New Guinea.^9^ The Lao PDR component was embedded within a prospective hospital-based study of the aetiology of ARI.^23^ We used the same terminology outlined by Halloran *et al* in which indirect effects are defined as the population-level effects of a vaccination strategy experienced by individuals regardless of vaccination status.^24^

### Participant recruitment

Between December 2013 and December 2019, study doctors recruited patients on weekdays from all paediatric wards of Mahosot Hospital. Eligible participants were children aged 2–59 months with ARI—defined as fever (self-reported or documented >38.0°C) and one or more respiratory symptoms and signs including dyspnoea, cough, rhinitis or abnormal pulmonary auscultatory examination, with symptom onset within 14 days prior to admission. Children that did not satisfy the eligibility criteria were excluded.

Following informed consent, NP samples were collected from cases using paediatric flocked swabs (Copan Diagnostics). Swab samples were stored in 1 mL skim milk tryptone-glucose-glycerol media, then transported to the local laboratory for handling and storage according to WHO guidelines.^25^ The majority of swabs were collected within 24 hours of admission to hospital (with the exception of admissions from Friday to Saturday which might be delayed for 1–3 days, since recruitment was during weekdays only). Data on demographics, medical care and hospital admission outcomes were collected for each participant. Individual PCV13 vaccination status was determined based on documented evidence of receiving an adequate number of PCV13 doses at least 14 days before enrolment.^26^ To determine vaccination status, we checked the parent-held MCH card for recorded PCV13 dates of administration or, if these were not available, study staff contacted the child’s health centre. A participant was defined as vaccinated if they had received an adequate number of doses for immune protection that is, two or more PCV13 doses at less than 12 months of age, or at least one dose at or after 12 months of age.^11^ Conversely, a participant was defined as ‘under-vaccinated’ if they had received less than the adequate number of PCV13 doses for immune protection, including those who had received no doses.^11^ For participants without written evidence of PCV13 status, we further classified them as under-vaccinated if parents verbally reported that the participant had not received any vaccines or if the participant was not age eligible to receive PCV13.

Participant data were double-entered using electronic databases (Microsoft Access and REDCap). Regular double-entry discrepancy and logic checks were conducted prior to analysis.

### Village vaccination coverage

Village vaccination coverage data were also collected to address aims 2 and 3. Visits to health centres for data collection were organised based on a list of villages where participants resided for participants enrolled up to June 2017. Visits commenced in June 2017 and were completed in June 2018. Therefore, analyses for aims 2 and 3 included all participants recruited up to June 2017 and the additional 77 participants enrolled up to June 2018 that resided in villages where vaccination data had already been collected. To determine the number of children vaccinated for PCV13 in each village, we extracted and transcribed relevant data from register books into a study database. Written register books contain line-listed data for each child residing in the village and the dates vaccinations were administered. Study staff also provided qualitative assessment of vaccination coverage data quality. Data were categorised as high quality if PCV13 dates were clearly identified and legible and registration books were available. For more details see online supplemental appendix page 2.

10.1136/bmjgh-2021-005187.supp1 Supplementary data

For each participant, we determined the village-level PCV13 coverage at the time each participant was enrolled—defined as the number of children under 5 years of age vaccinated in the participant’s village (at participant enrolment date) divided by the population of all children under 5 years of age in their village in 2015 (approximately midpoint of study period). Vaccine coverage was calculated among children less than 5 years of age, which includes children ineligible for PCV13 by age, since this is a critical age group primarily responsible for pneumococcal transmission.^20^ The number of children under 5 years of age living in each village was sourced from the health facility responsible for providing health services for each village. These population data were based on a government census conducted in 2015.^22^ We excluded Sisattanak district in Vientiane Capital, which has a population of 11 069 children under 5 years, where immunisation records were recorded by dose rather than by child, therefore we were unable to determine how many children were vaccinated according to our study definitions.

### Laboratory procedures

Samples were shipped on dry ice to the Murdoch Children’s Research Institute, Melbourne, Australia for sample testing and analysis. Samples were screened for the presence of pneumococci using quantitative real-time PCR (qPCR) targeting the *lytA* gene.^19^ Samples that were *lytA* qPCR positive (Ct value <35) or equivocal (Ct value 35–40) were cultured for molecular serotyping of pneumococcal isolates by microarray. Samples with Ct >40.0 were considered negative for pneumococcus. Molecular serotyping was performed using Senti-SP V.1.5 microarray (BUGS Bioscience) as previously described.^27 28^ Samples that were *lytA* qPCR positive (Ct value <35) but not able to be serotyped (either culture negative or due to repeated technical difficulties with DNA extraction) were considered pneumococcal positive, serotype unknown. Laboratory data were cleaned prior to analysis, including the removal of non-pneumococcal microarray calls. For more details on sample collection, transport, DNA extraction, *lytA* qPCR, culture and microarray methods, refer to previously published methods.^19^

As pneumococcal carriage prevalence and serotype distribution from hospital-based carriage surveillance may be affected by prior antibiotic use, we determined the presence of antimicrobial resistance (AMR) genes to enable comparison with other settings. The microarray detects 10 AMR genes associated with mobile genetic elements, encoding resistance to tetracycline (tetM, tetK, tetO, tetL), chloramphenicol (cat), macrolides (mefA, ermB, ermC), kanamycin (aphA3), streptothricin (sat4), lincosamides (ermB, ermC), and streptogramin B (ermB, ermC). As microarray currently provides a single overall AMR profile for all species detected, we restricted analysis to samples containing a single pneumococcal type with no other species identified, as per previous studies.^19^

Overall pneumococcal carriage was defined as detection of any pneumococcus in an NP swab sample (including samples that were *lytA* positive and culture negative (n=71) or were unable to be serotyped due to repeated technical difficulties with the DNA extraction (n=1)). VT carriage was defined as NP carriage of at least one pneumococcal serotype included in the PCV13 vaccine: serotypes 1, 3, 4, 5, 6A, 6B, 7F, 9V, 14, 18C, 19A, 19F and 23F. Samples were considered VT carriage positive if they contained at least one PCV13 serotype, regardless of any other serotypes present in the sample. Similarly, samples were considered non-VT carriage positive if they included at least one non-PCV13 serotype, regardless of any other serotypes present in the sample.

### Analyses

Characteristics of all the participants enrolled up to December 2019 were summarised by percentages, or medians and IQRs, where appropriate. We compared study characteristics by PCV13 vaccination status to identify any differences between vaccinated and undervaccinated children, as well as those with missing vaccination status. To show trends in VT carriage among vaccinated and under-vaccinated children over time (aim 1), we calculated moving carriage prevalence rates within 7-month rolling intervals to smooth random variation due to small monthly sample sizes. We included all participants with data on PCV13 status and adjusted the carriage prevalence rates by age group (2–11 months, 12–23 months, 24–59 months), using a direct adjustment method.^29^ We adjusted for age to account for random variation in the age of participants recruited each month, since age is a known determinant of pneumococcal carriage.

To describe the PCV13 coverage data, we tabulated the median village vaccination coverage by year. We also reported on the quality of vaccine coverage data by village. Annual village PCV13 coverage was mapped by coverage quintiles and an additional category for coverage above 100% (0–19,20-39,40–59,60-79,80–99,100+%) for Vientiane Province from 2014 to 2017 using QGIS V.3.8.^30^ Village vaccine coverage can appear to exceed 100% due to inaccuracies in population denominators or if children from outside the catchment area have received vaccinations at the health centre. Coverage data from 2018 were not mapped due to the small number of data points from seven villages only.

To examine the relationship between village-level PCV13 coverage and VT carriage (aim 2), we first calculated the PCV13 coverage in each participant’s village of residence on the date each participant was enrolled. To describe the data, we tabulated VT carriage prevalence among participants at quartiles of district-level PCV13 coverage (at the time of each participants’ enrolment) stratified by vaccination status.

For both adjusted and unadjusted (‘crude’) analyses examining the relationship between PCV13 coverage and VT carriage, we used generalised estimating equations with a binomial distribution, a logit link function and an exchangeable correlation structure, accounting for clustering at the village level, since participants from the same village are not independent. We included participants with complete data for all variables in the model (complete case analysis). We adjusted for PCV13 status, age in years, season (month of swab collection), monthly family income category (as a proxy for socioeconomic status), number of children under five in the household, and kindergarten attendance. These covariates were selected a priori using a directed acyclic graph, informed by relevant literature and refined through expert consultation (online supplemental figure 1). The linearity assumption was assessed using a lowess plot.

In order to quantify the effect of PCV13 coverage on VT carriage prevalence, we estimated and graphed marginal mean VT carriage prevalence by decile of PCV13 coverage, accounting for the balance of the other covariates across all the individuals, using the margins and marginsplot commands in Stata. To act as a bias indicator, we constructed an additional model with overall pneumococcal carriage as the outcome, since we do not expect that vaccine introduction will impact overall carriage prevalence. Many studies indicate that overall pneumococcal carriage remains constant following PCV introduction despite decreases in VT carriage due to corresponding increases in non-VT carriage prevalence.^9 15^

Since PCV13 status was included as a covariate in the model developed for aim 2, we were able use the same model output to calculate adjusted VE for aim 3. VE was calculated as one minus the the odds ratio of PCV13 carriage in vaccinated versus undervaccinated children multiplied by 100.

For aims 2 and 3, sensitivity analyses were performed, excluding participants with low quality vaccination coverage data, to determine whether results were affected by data quality.

All analyses were undertaken using Stata V.15.^31^ The community-contributed command baselinetable was used to construct table 1.^32^

**Table 1:** Participant characteristics by 13-valent pneumococcal conjugate vaccine (PCV13) status, Lao PDR, December 2013–June 2019

	Total	PCV13 vaccination status, n (%)*		
		Undervaccinated†	Vaccinated	Missing vaccination status
	N=1485*	N=492	N=757	N=236
**Median age in months, (IQR**)	15.0 (8.0–26.0)	18.0 (6.0–32.0)	14.0 (9.0–22.0)	18.0 (9.0–27.0)
**Age group, n (%**)				
**2–11 months**	590 (39.7)	198 (40.2)	314 (41.5)	78 (33.1)
**12–35 months**	466 (31.4)	102 (20.7)	285 (37.6)	79 (33.5)
**36–59 months**	429 (28.9)	192 (39.0)	158 (20.9)	79 (33.5)
**Male, n (%**)	825 (55.6)	261 (53.0)	441 (58.3)	123 (52.1)
**Year of enrolment, n (%**)				
**2013‡**	6 (0.4)	4 (0.8)	2 (0.3)	0 (0.0)
**2014**	353 (23.8)	219 (44.5)	105 (13.9)	29 (12.3)
**2015**	321 (21.6)	113 (23.0)	162 (21.4)	46 (19.5)
**2016**	286 (19.3)	73 (14.8)	155 (20.5)	58 (24.6)
**2017**	229 (15.4)	36 (7.3)	145 (19.2)	48 (20.3)
**2018**	164 (11.0)	33 (6.7)	109 (14.4)	22 (9.3)
**2019**	126 (8.5)	14 (2.8)	79 (10.4)	33 (14.0)
**Wet season, n (%**)	748 (50.4)	252 (51.2)	365 (48.2)	131 (55.5)
**Urban§, n (%) (N=1482**)	1413 (95.3)	465 (94.5)	731 (96.9)	217 (91.9)
**Kindergarten attendance, n (%) (N=1477**)	329 (22.3)	113 (23.3)	150 (19.8)	66 (28.1)
**Median no of other children <5 years in house, (IQR) (N=1476**)	1.0 (1.0–2.0)	1.0 (1.0–2.0)	1.0 (1.0–2.0)	1.0 (1.0–2.0)
**Family income per month, n (%) (N=1476**)				
**≤**₭**250 000**	74 (5.0)	47 (9.6)	20 (2.7)	7 (3.0)
₭**250 001–**₭**1 000 000 kip**	301 (20.4)	155 (31.6)	109 (14.5)	37 (15.9)
₭**1 000 001–**₭**3 000 000**	591 (40.0)	201 (41.0)	294 (39.0)	96 (41.4)
₭**3 000 001–**₭**5 000 000**	380 (25.7)	69 (14.1)	245 (32.5)	66 (28.4)
**>**₭**5 000 000**	130 (8.8)	18 (3.7)	86 (11.4)	26 (11.2)
**Maternal education (completion of primary school), n (%) (N=1345**)	1083 (80.5)	342 (73.2)	577 (85.9)	164 (79.6)
**Piped water source, n (%) (N=1483**)	843 (56.8)	245 (49.8)	467 (61.8)	131 (55.7)
**Wood or charcoal used for cooking fuel, n (%) (N=1178**)	1029 (87.4)	248 (81.8)	597 (89.6)	184 (88.0)
**Smoker in the house, n (%) (N=1471**)	632 (43.0)	200 (41.6)	321 (42.5)	111 (47.2)
**Pneumonia categories, n (%)¶ (N=1467**)				
**Acute respiratory infection** (**not pneumonia**)	574 (39.1)	180 (36.9)	298 (39.8)	96 (41.7)
**Pneumonia (not severe**)	417 (28.4)	127 (26.0)	222 (29.6)	68 (29.6)
**Severe pneumonia**	476 (32.4)	181 (37.1)	229 (30.6)	66 (28.7)
Pre-admission antibiotics**, n (%) (N=1432)	766 (53.5)	264 (55.6)	397 (54.0)	105 (47.3)
**Received antibiotics in hospital, n (%) (N=1453**)	1266 (87.1)	430 (88.8)	638 (86.1)	198 (86.8)
**Comorbidities, n (%**)	165 (11.1)	51 (10.4)	87 (11.5)	27 (11.4)
**Pneumococcal carriage prevalence††, n (%**)	532 (35.8)	180 (36.6)	261 (34.5)	91 (38.6)
**Vaccine-type pneumococcal carriage prevalence††, n (%) (N=1415**)	188 (13.3)	87 (18.8)	70 (9.6)	31 (13.7)
**Non-vaccine-type pneumococcal carriage prevalence††, n (%) (N=1415**)	302 (21.3)	77 (16.7)	170 (23.4)	55 (24.3)
**Percentage of *lytA* positive samples, serotype unknown‡‡, n (%) (N=532**)	462 (86.8)	150 (83.3)	231 (88.5)	81 (89.0)
**Percentage of pneumococcal single-serotype samples with at least one AMR gene, n (%) (N=394**)	293 (74.4)	100 (78.7)	137 (69.2)	56 (81.2)

*N=1485 unless otherwise specified in the first column.

†Undervaccinated defined as less than one dose received under 12 months of age and no doses ≥12 months of age.

‡Recruitment started in December 2013.

§Categorised as urban if the village falls within Vientiane Capital.

¶WHO 2013 definition.

**Parent report.

††Pneumococcal carriage was defined as detection of any pneumococcus, including samples that were l*ytA* positive and serotype unknown. However, *lytA* positive samples with an unknown serotype were excluded from VT and NVT carriage prevalence estimates.

‡‡Serotypes were either unknown due to the sample being culture negative (n=71) or unable to be serotyped due to repeated technical difficulties with the DNA extraction (n=1)).

Lao PDR, Lao People’s Democratic Republic.

### Sample size

The sample size calculation was based on our primary question regarding the association between village PCV13 coverage and the VT carriage (aim 2), and was previously published in our study protocol.^9^ Calculations were performed using nQuery Advisor+nTerim V.4.0. Calculations were based on sample size methods for logistic regression models with a continuous covariate (ie, PCV13 coverage) and additional covariates, with inflation to account for clustering within villages (intraclass coefficient of 0.1). At the mean level of PCV13 coverage, we assumed a VT carriage prevalence of 30%, while at a coverage level one SD above the mean, we assumed VT carriage would decline to 20%. Assuming a significance level of 0.05, allowing for adjustment using multiple covariates with an R-squared value of 0.4, 600 participants would provide 87% power to answer the primary question.

## Results

### Participant characteristics

**Figure 1:**
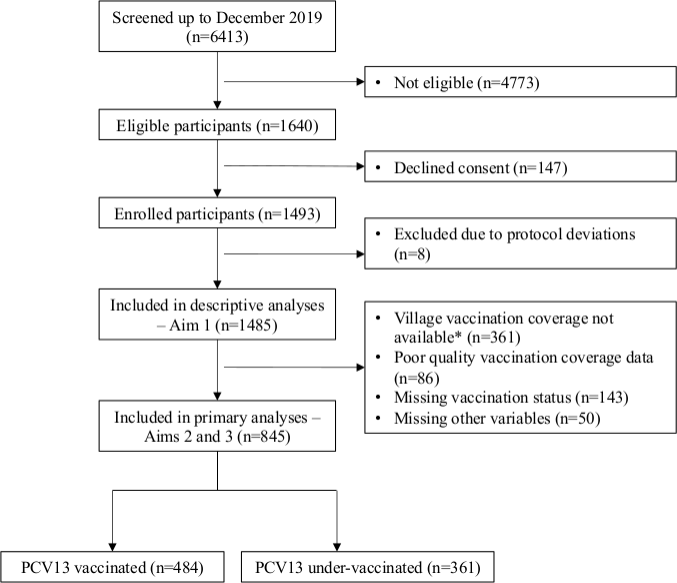
Flow chart of study recruitment at Mahosot Hospital, Lao PDR, December 2013–June 2019. *Final rounds of vaccine coverage data collection for PCV13 vaccination coverage were conducted up to June 2018. Lao PDR; Lao People’s Democratic Republic; PCV; pneumococcal conjugate vaccine.

Between December 2013 and December 2019, 6413 children were screened for eligibility. We enrolled 1493 and included 1 485 in our descriptive analyses of carriage trends over time (figure 1).

Table 1 summarises the characteristics of the study participants by PCV13 vaccination status. The percentage of missing data was less than 10% for all variables except PCV13 status (15% missing). The characteristics of those with missing PCV13 status were largely similar to the overall participant cohort, with minor differences in the percentage of participants with a smoker in the house, preadmission antibiotic use and AMR gene patterns. There were no differences in antibiotic use or the percentage of *lytA* positive samples that cultured pneumococci by PCV13 vaccination status. There were minor differences in the other characteristics of participants based on their vaccination status (table 1), for example, level of maternal education, indicated by completion of primary school, was higher in vaccinated children (85.9%) compared with undervaccinated children (73.2%). VT carriage prevalence and percentage of pneumococcal single-serotype samples with at least one AMR gene were both lower among vaccinated participants.

### Pneumococcal carriage

Among the 1485 participants, pneumococcal carriage prevalence was 35.8%. Eighty-seven per cent (462/532) of *lytA* positive samples were successfully cultured for pneumococci, of which 40.7% contained PCV13 serotypes. The individual serotypes identified are graphed by year in online supplemental figure 2. AMR genes were common, with 74.4% of pneumococcal positive single-serotype samples containing at least one of the 10 AMR genes assessed. Overall carriage and AMR gene patterns were largely consistent over time and VT carriage prevalence decreased over time (table 2). There appeared to be an increase in the number of samples that were *lytA* positive and culture negative over time, which was not explained by changes in antibiotic usage or severity of illness among the participants over time (online supplemental table 1).

**Table 2:** Characteristics of pneumococcal carriage samples by year, Vientiane, Lao PDR, December 2013–June 2019

	2013–2014	2015	2016	2017	2018	2019
Pneumococcal carriage prevalence*, n/N (%)	156/359 (43.5)	117/321 (36.4)	90/286 (31.5)	81/229 (35.4)	42/164 (25.6)	47/126 (37.3)
Vaccine-type (VT) pneumococcal carriage prevalence*, n/N (%)	81/344 (23.6)	47/309 (15.2)	29/275 (10.6)	26/217 (12.0)	5/154 (3.3)	0/116 (0.0)
Non-vaccine-type pneumococcal carriage prevalence*, n/N (%)	70 (20.3)	67 (21.7)	54 (19.6)	47 (21.7)	27 (17.5)	37 (31.9)
**Percentage of *lytA* positive samples, serotype unknown†, n/N (%**)	15/156 (9.6)	12/117 (10.3)	11/89 (12.4)	12/81 (14.8)	10/42 (23.8)	10/47 (21.3)
**Percentage of pneumococcal single-serotype samples with at least one AMR gene, n/N (%**)	95/127 (74.8)	61/86 (70.9)	49/66 (74.2)	47/57 (82.5)	22/28 (78.6)	19/30 (63.3)

*Pneumococcal carriage was defined as detection of any pneumococcus, including samples that were *lytA* positive and serotype unknown. However, *lytA* positive samples with an unknown serotype were excluded from VT and NVT carriage prevalence estimates.

†Serotypes were either unknown due to the sample being culture negative (n=71) or unable to be serotyped due to repeated technical difficulties with the DNA extraction (n=1)).

Lao PDR, Lao People’s Democratic Republic.

**Figure 2:**
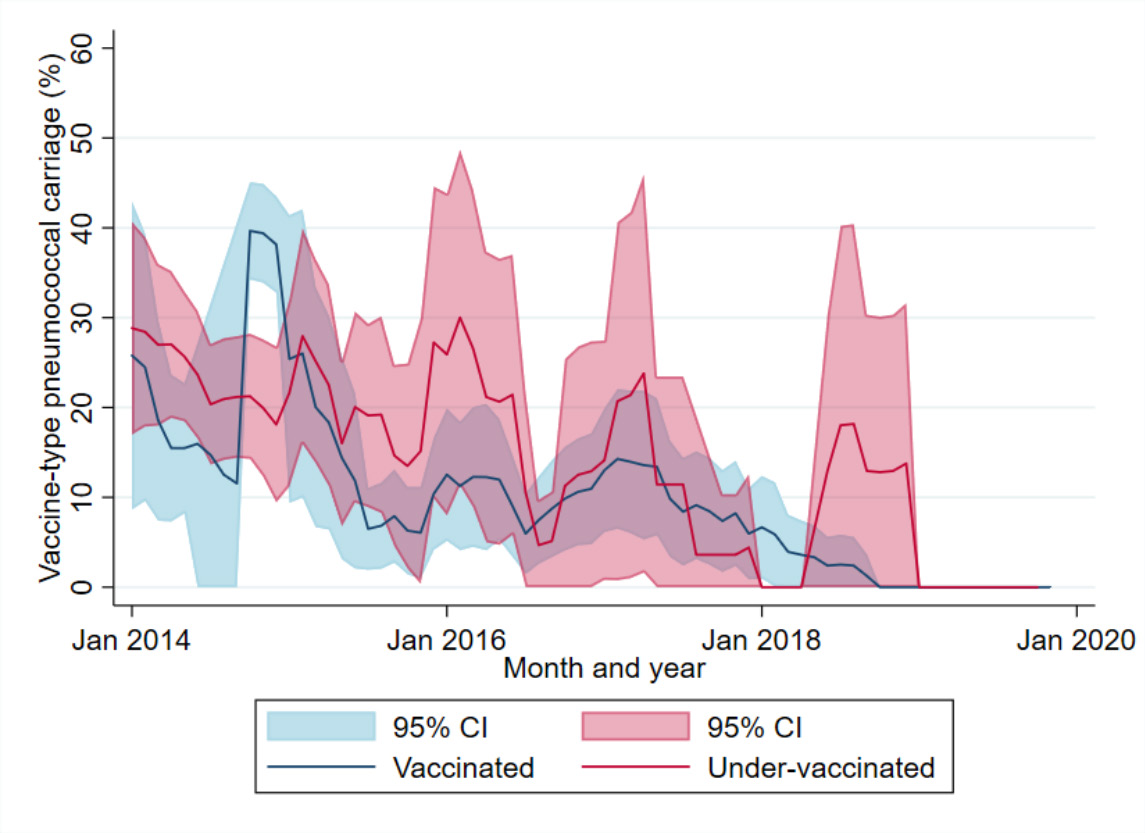
Monthly adjusted prevalence of vaccine-type carriage (7-month rolling intervals), adjusted by age group, among children 2–59 months of age admitted with acute respiratory infection, by PCV13 status, Vientiane, Lao PDR, December 2013–December 2019. Lao PDR; Lao People’s Democratic Republic; PCV; pneumococcal conjugate vaccine.

The adjusted monthly prevalence of VT carriage following PCV13 introduction is shown in figure 2. Over this period, the adjusted VT carriage rates declined in both vaccinated and under-vaccinated participants (figure 2).

### PCV coverage

**Figure 3:**
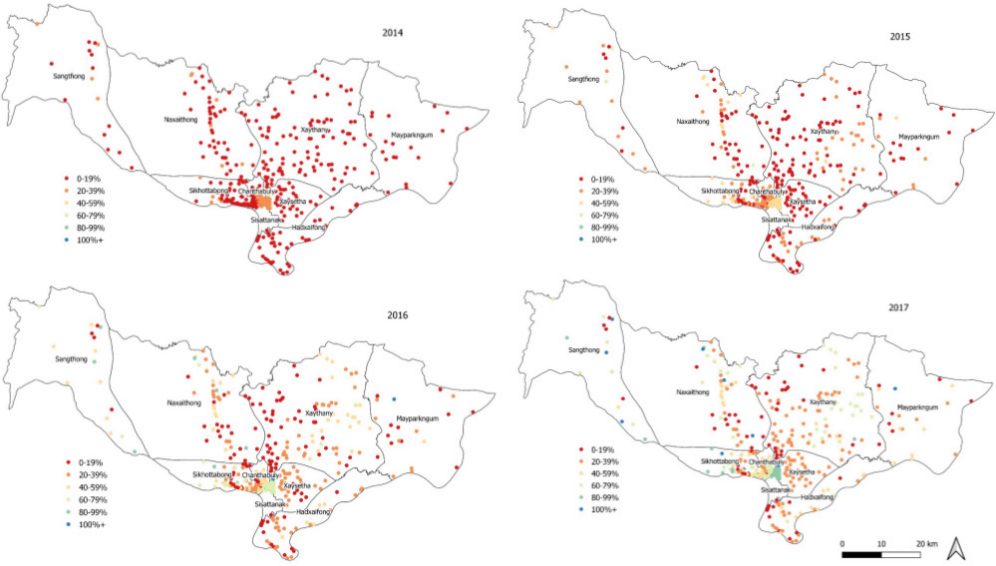
Map of 13-valent PCV13 coverage among children under 5 years of age by village, Vientiane Capital*, Lao PDR, 2014–2017; *Excluding Sisattanak district; data from 2018 was excluded due to small numbers of villages. Lao PDR; Lao People’s Democratic Republic; PCV; pneumococcal conjugate vaccine.

Vaccination and census data from 590 villages, comprising 1 549 534 children under 5 years of age, were collected. Vaccination coverage data were assessed as high quality for 69 villages (11%), with PCV13 clearly marked and dates being legible for 189 villages (32%) and no evidence of missing vaccination books for 456 villages (77%). From 2014 to 2017, the median PCV13 coverage among all villages (excluding Sisattanak district) increased from 4.5% to 37.5% among children under 5 years (online supplemental tables 2 and 3). Figure 3 shows the heterogeneity in coverage among children under five in each participant’s village within Vientiane Capital. Maps of village level PCV13 coverage for participants from outside of Vientiane Capital are presented in online supplemental figure 3.

### Association between PCV13 coverage and VT carriage

VT carriage prevalence among participants stratified by levels of PCV13 coverage indicated an inverse relationship between PCV13 coverage and VT carriage among under-vaccinated participants (table 3). Analyses were based on 845 participants from 368 villages, with a population of 269 863 children under five years of age.

**Table 3:** Vaccine-type pneumococcal carriage by quartile of village 13-valent pneumococcal conjugate vaccine (PCV13) coverage, Lao PDR, December 2013–June 2018*

	Vaccine-type pneumococcal carriage		
Levels of PCV13 coverage	All children n/N (%)	Vaccinated children n/N (%)	Undervaccinated children n/N (%)
**<25%**	101/500 (20.2)	36/243 (14.8)	65/257 (25.3)
**25%–50%**	23/212 (10.9)	13/139 (9.3)	10/73 (13.7)
**50%–75%**	12/85 (14.1)	8/65 (12.3)	4/20 (20.0)
**>75%**	3/48 (6.3)	3/37 (8.1)	0/11 (0.0)

*Includes all participants recruited up to June 2017 and the additional 77 participants enrolled up to June 2018 that resided in villages where vaccination data had already been collected.

Lao PDR, Lao People’s Democratic Republic; PCV, pneumococcal conjugate vaccine.

**Figure 4:**
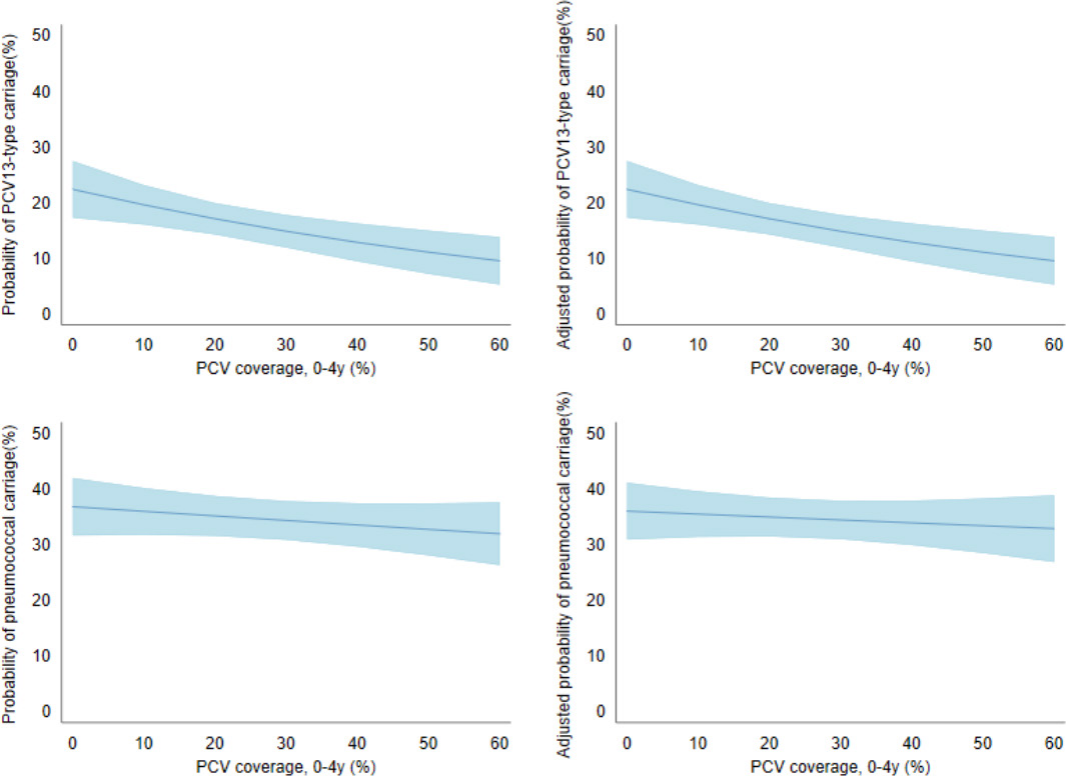
Predicted probability of vaccine-type carriage (top row) and overall pneumococcal carriage (bottom row) by level of 13-valent pneumococcal conjugate vaccine (PCV13) coverage, Lao PDR, 2013–2018; figures on the left are unadjusted while figures on the right are adjusted by vaccination status, family income, age, season, number of children under 5 in the household and kindergarten attendance. Lao PDR; Lao People’s Democratic Republic.

From the adjusted analyses, for each increase in percentage point of PCV13 coverage, the estimated odds of VT carriage decreased by 1.1% (95% CI 0.0% to 2.2%; p=0.056) (table 3). As expected, there was no evidence of an association between PCV13 coverage and overall pneumococcal carriage after adjustment (OR 0.998 (95% CI 0.991 to 1.004); p=0.475) (table 4). The predicted probability of VT carriage at each decile of village PCV13 coverage up to 60% is shown in figure 4. We estimated up to 60% coverage, since there were few participants from villages with greater than 60% coverage among children under five. As PCV13 coverage increased from zero to 60%, VT carriage prevalence reduced by 36%, from 20.0% (95% CI 15.2% to 24.9%) to 12.8% (95% CI 8.5% to 17.1%). From this model, which includes PCV13 status, the adjusted VE was 38.1% (95% CI 4.1 to 60.0; p=0.032) against VT carriage, adjusting for PCV13 coverage (indirect effects), family income, age, season, number of children under five in the household and kindergarten attendance. The sensitivity analysis which excluded participants from villages with poor quality vaccination coverage data found similar effect sizes for both direct and indirect effects of PCV13 (online supplemental table 4).

**Table 4:** Crude and adjusted* ORs of vaccine-type and overall pneumococcal carriage among children under 5, by percentage increase in 13-valent pneumococcal conjugate vaccine (PCV13) coverage and individual vaccination status, Lao PDR, December 2013–June 2018†; results are shown for complete participant analyses (n=845)

	Crude		Adjusted*	
	OR (95% CI)	P value	OR (95% CI)	P value
**Vaccine-type (VT) carriage**				
**PCV13 coverage**	0.983 (0.972 to 0.994)	0.003	0.989 (0.978 to 1.000)	0.056
**PCV13 status**	0.520 (0.356 to 0.759)	0.001	0.619 (0.400 to 0.959)	0.032
**PCV13 effectiveness (%)‡**	48.0 (34.1 to 64.1)	0.001	38.1 (4.1 to 60.0)	0.032
**Overall pneumococcal carriage**				
**PCV13 coverage**	0.996 (0.990 to 1.003)	0.255	0.998 (0.991 to 1.004)	0.475
**PCV13 status**	1.026 (0.778 to 1.356)	0.859	1.094 (0.777 to 1.542)	0.606
**PCV13 effectiveness (%)‡**	−2.6% (−35.6 to 22.2)	0.859	−9.4 (-54.2 to 22.3)	0.606

*Adjusted by family income, age, season, number of children under 5 in the household and kindergarten attendance.

†Includes all participants recruited up to June 2017 and the additional 77 participants enrolled up to June 2018 that resided in villages where vaccination data had already been collected.

‡PCV13 effectiveness against VT carriage was one minus the adjusted OR for the association between VT carriage and individual PCV13 status and multiplied by 100.

PCV, pneumococcal conjugate vaccine.

## Discussion

Using a novel approach, our study is the first to show substantial indirect effects on pneumococcal VT carriage in a setting with moderate and heterogeneous PCV13 coverage. We estimated a 36% reduction in VT carriage through indirect effects alone up to 5 years following PCV13 introduction, as coverage increased from zero to 60%. We expect that reductions in VT carriage in Lao PDR will equate to reductions in VT disease, since carriage is a prerequisite for disease.^5^ Our findings are consistent with recent community carriage surveys conducted in Lao PDR which found reductions, although non-statistically significant, in VT carriage among infants too young to be vaccinated only 2 years following PCV introduction (ie, indirect effects).^33^ Our results are also in line with previous studies from the USA, reporting that moderate coverage among children under 5 years of age (58%–75% coverage) was sufficient to induce substantial indirect effects on VT carriage.^11 12^ Previous studies have demonstrated a relationship between vaccine coverage and indirect effects, including a meta-analysis of studies from middle and high-income countries that reported a 5% decline in pneumococcal disease (95% credible interval 2%–11%) for each 10% increase in coverage.^4 34^

Our findings also provide the first estimates of VE against VT carriage (ie, direct effects) for PCV13 in Asia among children 2–59 months of age, adding support to a previous community carriage study from Lao PDR which demonstrated a 23% reduction in VT carriage following PCV introduction among 12–23 month old children.^33^ Importantly, both estimates accord with VE estimates against a disease outcome in Lao PDR: among the same cohort as this study, we found an adjusted PCV13 effectiveness of 37% against hypoxic pneumonia among children under five.^35^ A study from Brazil reported a similar VE with three primary doses of PCV13 at 44.0% (14.2%–63.5%) against VT carriage among children 7–11 months of age,^36^ while a study from Israel found a VE of 62% (95% CI 33% to 83%) against PCV13 carriage among children 25–59 months of age.^37^ Our results were also lower than estimates of VE for PCV13 from modelling in Malawi, at 66.87% (95% CI 50.49% to 82.26%).^38^ The differing estimates may relate to the timing of assessment after vaccine is administered to each child (ie, waning direct effects), since a meta-regression of original efficacy results from PCV7 trials reported VEs ranging from 62% (95% CI 52% to 72%) 4 months after vaccination to 42% (95% CI 19% to 54%) 5 years after vaccination.^39^

At 5 years following PCV introduction, VTs were no longer detected among a total of 116 samples—indicating elimination or near-elimination of VTs in Lao PDR. This is consistent with high-income countries, such as the USA and England, which observed declines of VT carriage to less than 5% at 8 and 6 years following vaccine introduction.^40 41^However, we recommend continued surveillance to determine whether this finding is sustained, especially since enrolment declined overtime.

Our carriage surveillance offers a novel approach to monitor VT carriage in resource-limited settings.^10^ In addition to demonstrating declines in VT carriage among both vaccinated and under-vaccinated participants, our carriage surveillance indicates that pneumococcal carriage in Lao PDR may be seasonal. Previous studies have also demonstrated variations in pneumococcal carriage prevalence by season in a range of settings.^42 43^ Carriage surveillance can help identify when VTs are under control, and therefore a 1+1 schedule switch can be considered, as well as continue to monitor VT carriage after any changes to the vaccination programme. This study design has both advantages and disadvantages. Compared with recruiting healthy children from the community, children in hospitals are an accessible population which is more feasible to sample in low-resource settings. However in this patient population, the use of antibiotics can reduce detection of NP pneumococcal carriage or select for carriage of antibiotic resistant strains of particular serotypes.^29^ We have shown that, despite high levels of antibiotic use in our study cohort, 87.4% of *lytA* positive samples were able to be cultured for molecular serotyping. The percentages of culture-positive samples were similar by vaccination status and remained consistent over time, indicating that prior antibiotic use is unlikely to be a major confounder. The AMR prevalence in our sample (74.4%) was similar to community carriage surveys conducted in Lao PDR over a similar period (70.8%), suggesting that the pneumococcal epidemiology in our hospitalised cohort reflects community trends.^33^ Similarly, our estimates of overall carriage and VT carriage prevalence from 2014 were also comparable to these survey estimates (14.3% overall and 6.5% VT carriage prevalence among infants (5–8 weeks); 55.8% overall and 32.9% VT carriage prevalence among children (12–23 months)).^19^ Therefore, we suggest that hospital-based surveillance can provide useful indication of pneumococcal trends in the community, although we anticipate that our results are more reflective of pneumococcal serotypes causing disease.^29^ Further research comparing hospital-based and community-based carriage studies are required to understand the generalisability of hospital-based surveillance.

A strength of our study is the use of vaccine coverage at a village level as an exposure to determine vaccine impacts. This is a novel application of methods originally used to evaluate cholera vaccines,^9^ enabling us to determine robust estimates for both direct and indirect effects of PCV13 within the same model.^44^ We also assessed the likelihood of bias, finding no association between PCV coverage and overall pneumococcal carriage. Since most studies show that PCV does not reduce overall pneumococcal carriage,^10^ the contrasting results, with the presence of an association with VT carriage but not overall carriage, support the validity of our findings attributing reductions in VT carriage to vaccine coverage rather than other confounding factors. Our estimates of vaccine coverage were based on administrative data, which have previously been reported to overestimate coverage compared with survey data.^45 46^ There may also be variation in data quality by health centre, errors in transcribing hand-written registers and mismatches in numerators and denominators for village-level estimates.^47^ However, our coverage results among children 12–23 months of age (59.7%, online supplemental table 4) were very similar to those from the Lao Social Indicator Survey from 2017 which found that coverage of two doses of PCV was 53% among children 12–23 months of age in Vientiane Capital, our study site.^48^

A limitation of our study is that we did not assess carriage in adults and the elderly, populations which have been the greatest beneficiary of indirect effects in high-income countries. However, we expect that the indirect effects seen in children can be extrapolated to adults, since multiple studies from other settings with low pneumococcal carriage prevalence identify children under 5 years of age as the main drivers of transmission.^20 49 50^ Individual vaccination status was missing for 15% of our participants, however, comparison of participant characteristics in table 1 suggests no systematic differences between groups included and excluded from the primary analyses. Lastly, within our study period, median vaccine coverage reached only 37.5% (IQR 24.1%–56.4)% among all children under 5 years of age. Furthermore, there were fewer participants, including participants carrying VTs, recruited in 2018 and 2019, as coverage increased. Therefore, we have only estimated indirect effects up to 60% coverage, although we expect continued increases in indirect effects as coverage increases.

The degree of indirect effects has two important policy implications. First, the cost-effectiveness of the vaccine greatly improves when the indirect effects are present and taken into account, helping policy makers to justify the public funding of the vaccine.^51^ Second, the control of VT transmission is a necessary prerequisite for the use of 1+1 schedules, which are currently under investigation, and has the potential to maintain vaccine impacts through indirect effects while reducing programme costs.^8^ The generation of indirect effects is an important component of achieving control of VTs. Furthermore the success of this schedule relies on the ongoing generation of indirect effects to protect children under the age of 12 months who do not have sufficient individual immunity to protect them from disease.^52^

Our results provide insight into the direct and indirect effects of PCV in Lao PDR and have important implications for other countries in the region, many of which have yet to introduce PCV into routine childhood immunisation schedules. Importantly our results demonstrate that near elimination of VT carriage is possible in LMICs, and therefore, the introduction of cost-saving 1+1 schedules should be considered and evaluated for such settings. However, given the heterogeneity in vaccine coverage, it would be important to consider the possibility of continued pockets of VT pneumococcal transmission within areas of low vaccine coverage. We recommend continued pneumococcal carriage surveillance to ensure VTs remain under control among populations most at risk of disease.

## Data Availability

Anonymised data are available on request to FR (fmruss@unimelb.edu.au) for research purposes only and subject to approval from the Lao PDR Ministry of Health National Ethics Committee for Health Research.
