# Supplementary material for: Indirect effects of 13-valent pneumococcal conjugate vaccine on pneumococcal carriage in children hospitalised with acute respiratory infection despite heterogeneous vaccine coverage: an observational study in Lao People’s Democratic Republic
Source: BMJ Glob Health. 2021 Jun 9;6(6):e005187. doi: 10.1136/bmjgh-2021-005187 (PMC8191607; doi:10.1136/bmjgh-2021-005187)
Supplement: Supplementary data [file bmjgh-2021-005187supp001.pdf]

Supplementary appendix

Health centre data collection

Data from health centre immunisation register books were collected to determine the numerator for PCV13 coverage in each village. Study staff first generated a list of villages in which enrolled cases resided and determined the health centre that served each village. They then planned a schedule of health centre visits and prepared letters to each health centre detailing our study, the purpose of the visit, the month of the visit and contact details.

At each health centre, with the exception Sisattanak district, immunisations were recorded in register books which contain line-listed data of each child residing in the village and the dates that vaccinations were administered for that child. As with the cases, we defined a child as vaccinated if they had received two or more PCV13 doses at less than 12 months of age, or at least one dose at or after 12 months of age. In Sisattanak district, immunisations were recorded on sheets of paper by date of administration, with a new sheet for each day. We therefore excluded Sisattanak district, which has a population of 11 069 children under five years, from our analysis as we were unable to determine how many children were vaccinated according to our study definitions.

At each health centre visit, study staff took photographs of each page of each immunisation register book to facilitate transcription of hand-written data into a study database following the visit. To ensure accuracy of data transcription, study staff confirmed with health centre staff when PCV was introduced at the health centre and how PCV data was recorded. If the health centre transitioned between register books during the study period, staff clarified whether entries were transferred between old and new immunisation register books.

For each village, study staff assessed data quality according to three key measures:

1. Were PCVs clearly marked? – in earlier iterations of data registration books, there may not be a column a separate column to record PCV13 so health centre staff recorded doses of PCV13 in various ways
2. Were PCV13 dates legible?
3. Is there evidence of missing registration books?

Health centre data were categorised as low quality if PC13 were not clearly marked, or PCV dates were illegible or there was evidence of missing registration books.

Study staff transcribed the following data from register books into a study database: child's name, date of birth, village, and the date PCV13 was administered. To ensure that children were not entered into the database twice, study staff reviewed books for duplicate entries and crossed-out and highlighted records for entry. Where date of birth was not documented, dates of hepatitis B or BCG vaccination were used as surrogates as these vaccines are given at birth. Data were initially entered into an EpiData database and

transitioned to a RedCAP database in 2019. Following data entry, records were checked for logic and duplicates. We also conducted a random audit of 10% of entries to ensure data accuracy.

Data were entered for 53 672 children who received at least one dose of PCV13. Date of birth were missing 171 (0.3%) of children. Where date of birth was not known, we considered the child vaccinated only if they had received at least two doses of vaccine, since we were not able to determine whether they had received their first dose at greater than or equal to 12 months of age. If a child's records indicate receipt of the second or third dose of a vaccine, we assumed that earlier vaccines in the sequence had been given.

Variable selection using directed acyclic graphs (DAGs)

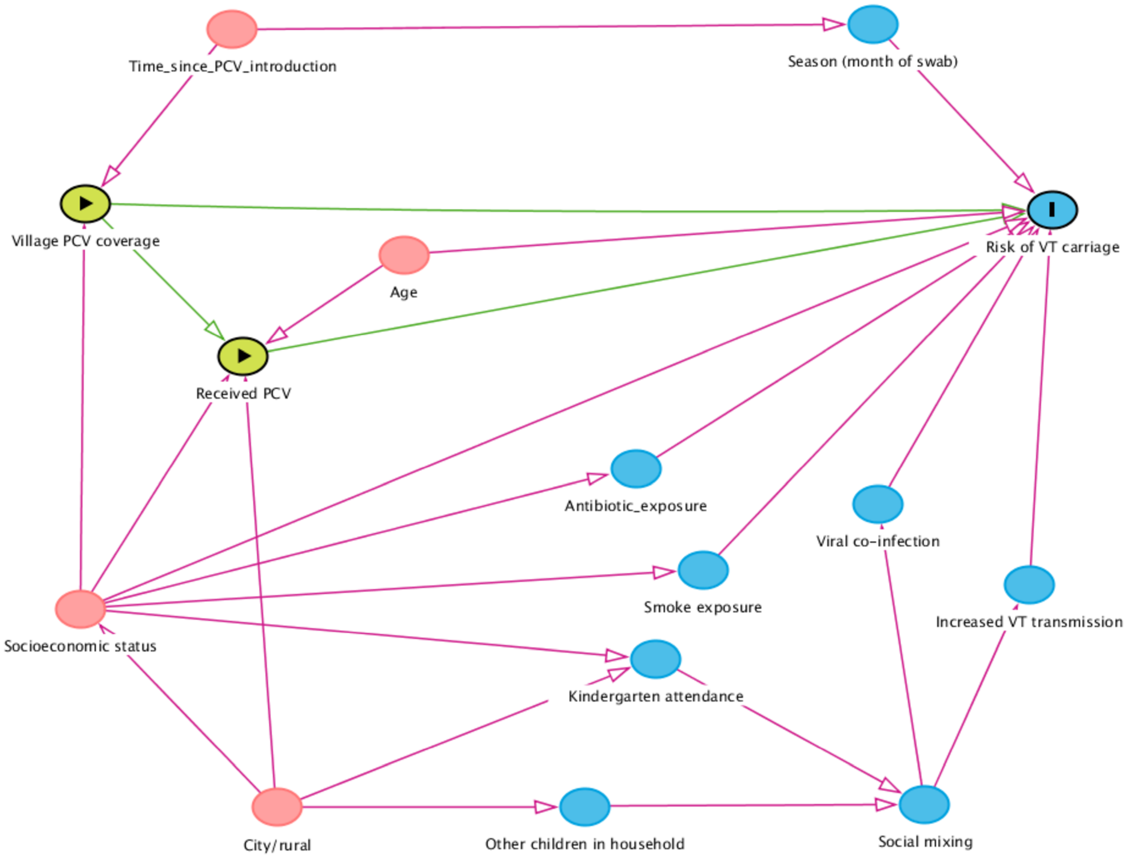

Supplementary figure 1: Directed acyclic graph of the association between the two exposures, village-level PCV13 coverage and PCV receipt and risk of VT carriage (outcome)

With directed acyclic graphs (DAG), arrows are used to indicate causal pathways between exposure and outcome.[1,2] The green lines highlight the causal relationship under investigation and the pink lines highlight potential biasing pathways. The blue variables are ancestors of the outcome, i.e. they directly or indirectly cause the outcome. While red variables are ancestors of both exposure and outcome. The diagram includes

potential confounders including both measured and unmeasured variables. Based on this diagram, we identified that adjusting for age, season, other children in the household, socioeconomic status and kindergarten attendance is sufficient to block biasing pathways. There may be time trends associated with risk of VT carriage, however since PCV coverage and time are collinear for the duration of our study, and we do not have any pre-PCV data, we were not able to include time in our analyses.

Supplementary table 1: Participant characteristics by year of enrolment, Lao PDR, December 2013 – June 2019

	2013-4	2015	2016	2017	2018	2019
**Median age in months, (IQR)**	14.0 (7.0-23.0)	15.0 (8.0-25.0)	16.0 (9.0-26.0)	15.0 (8.0-27.0)	17.5 (9.0-28.0)	17.0 (9.0-27.0)
**Age group, n (%)**						
**2-11m**	150 (41.8%)	131 (40.8%)	109 (38.1%)	95 (41.5%)	57 (34.8%)	48 (38.1%)
**12-35m**	121 (33.7%)	105 (32.7%)	94 (32.9%)	60 (26.2%)	50 (30.5%)	36 (28.6%)
**36-59m**	88 (24.5%)	85 (26.5%)	83 (29.0%)	74 (32.3%)	57 (34.8%)	42 (33.3%)
**Male, n (%)**	205 (57.1%)	181 (56.4%)	159 (55.6%)	128 (55.9%)	85 (51.8%)	67 (53.2%)
**Wet season, n (%)**	210 (58.5%)	124 (38.6%)	168 (58.7%)	105 (45.9%)	84 (51.2%)	57 (45.2%)
**Urban[§], n (%) (N=1482)**	346 (96.4%)	301 (94.1%)	268 (94.4%)	220 (96.1%)	158 (96.3%)	120 (95.2%)
**Kindergarten attendance, n (%) (N=1477)**	63 (17.8%)	62 (19.5%)	75 (26.2%)	52 (22.7%)	40 (24.4%)	37 (29.4%)
**Median number of other children <5y in house, (IQR) (N=1476)**	1.0 (1.0-2.0)	1.0 (1.0-2.0)	1.0 (1.0-2.0)	1.0 (1.0-2.0)	1.0 (1.0-2.0)	1.0 (1.0-2.0)
**Family income per month, n (%) (N=1476)**						
**< =250,000 kip**	64 (18.1%)	7 (2.2%)	3 (1.0%)	0 (0.0%)	0 (0.0%)	0 (0.0%)
**250,001 – 1,000,000 kip**	162 (45.8%)	99 (31.0%)	37 (12.9%)	1 (0.4%)	1 (0.6%)	1 (0.8%)
**1,000,001 – 3,000,000 kip**	93 (26.3%)	163 (51.1%)	140 (49.0%)	93 (41.0%)	53 (32.3%)	49 (38.9%)
**3,000,001 – 5,000,000 kip**	23 (6.5%)	43 (13.5%)	87 (30.4%)	97 (42.7%)	58 (35.4%)	72 (57.1%)
**>5,000,000 kip**	12 (3.4%)	7 (2.2%)	19 (6.6%)	36 (15.9%)	52 (31.7%)	4 (3.2%)
**Maternal education (completion of primary school), n (%) (N=1345)**	291 (82.4%)	251 (79.9%)	232 (82.0%)	59 (54.1%)	139 (86.9%)	111 (88.1%)

Chan J, *et al. BMJ Global Health* 2022; 6:e005187. doi: 10.1136/bmjgh-2021-005187

**Piped water source, n (%) (N=1483)**	201 (56.1%)	177 (55.3%)	173 (60.5%)	127 (55.5%)	94 (57.3%)	71 (56.3%)
**Wood or charcoal used for cooking fuel, n (%) (N=1178)**	5 (9.3%)	300 (93.8%)	257 (90.2%)	211 (92.1%)	147 (89.6%)	109 (86.5%)
**Smoker in the house, n (%) (N=1471)**	151 (43.4%)	121 (37.9%)	115 (40.4%)	122 (53.3%)	76 (46.3%)	47 (37.3%)
**Pneumonia categories, n (%)¶ (N=1467)**						
**Acute respiratory infection**	134 (37.3%)	130 (41.7%)	113 (40.1%)	94 (41.8%)	54 (33.1%)	49 (38.9%)
**Pneumonia (not severe)**	88 (24.5%)	98 (31.4%)	76 (27.0%)	62 (27.6%)	51 (31.3%)	42 (33.3%)
**Severe pneumonia**	137 (38.2%)	84 (26.9%)	93 (33.0%)	69 (30.7%)	58 (35.6%)	35 (27.8%)
**Pre-admission antibiotics**, n (%) (N=1432)**	225 (64.1%)	162 (51.8%)	114 (41.5%)	112 (53.8%)	91 (56.9%)	62 (49.6%)
**Received antibiotics in hospital, n (%) (N=1453)**	311 (87.1%)	272 (86.9%)	246 (88.8%)	193 (86.5%)	140 (87.0%)	104 (85.2%)
**Comorbidities, n (%)**	33 (9.2%)	7 (2.2%)	6 (2.1%)	42 (18.3%)	50 (30.5%)	27 (21.4%)
**Vaccination status, n(%) (N=1249)**						
**Under-vaccinated**	223 (62.1%)	113 (35.2%)	73 (25.5%)	36 (15.7%)	33 (20.1%)	14 (11.1%)
**Vaccinated**	107 (29.8%)	162 (50.5%)	155 (54.2%)	145 (63.3%)	109 (66.5%)	79 (62.7%)
**Missing**	29 (8.1%)	46 (14.3%)	58 (20.3%)	48 (21.0%)	22 (13.4%)	33 (26.2%)

*N=1485 unless otherwise specified in the first column; § Categorised as urban if the village falls within Vientiane Capital; ¶ WHO 2013 definition; **Parent report

Pneumococcal serotypes by year

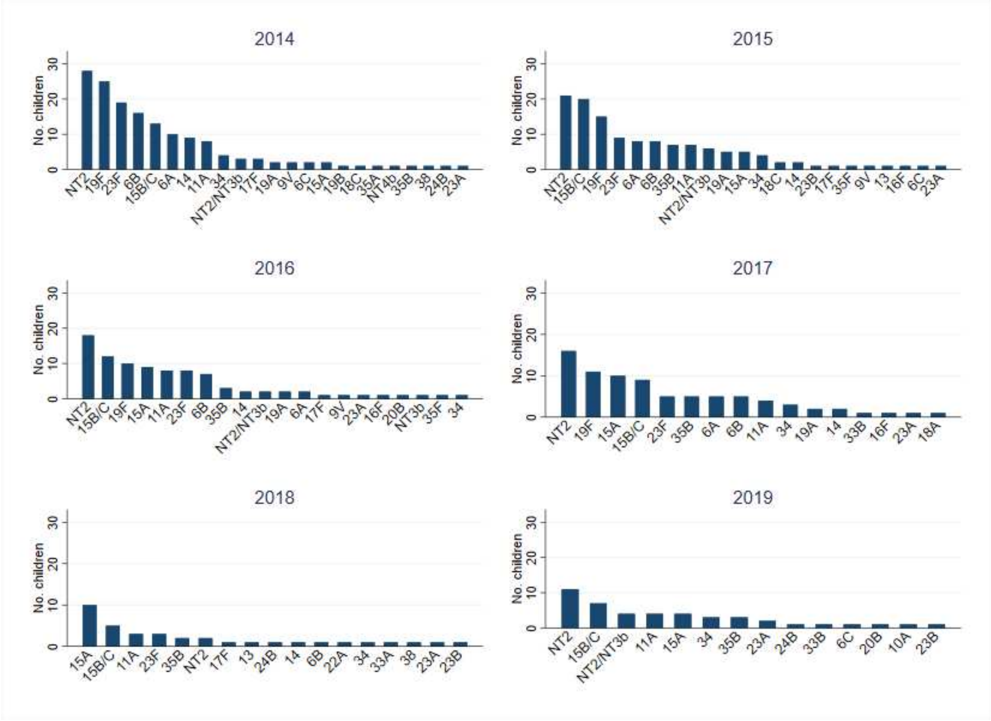

Supplementary figure 2: Pneumococcal serotypes by year, Lao PDR, 2014-2019

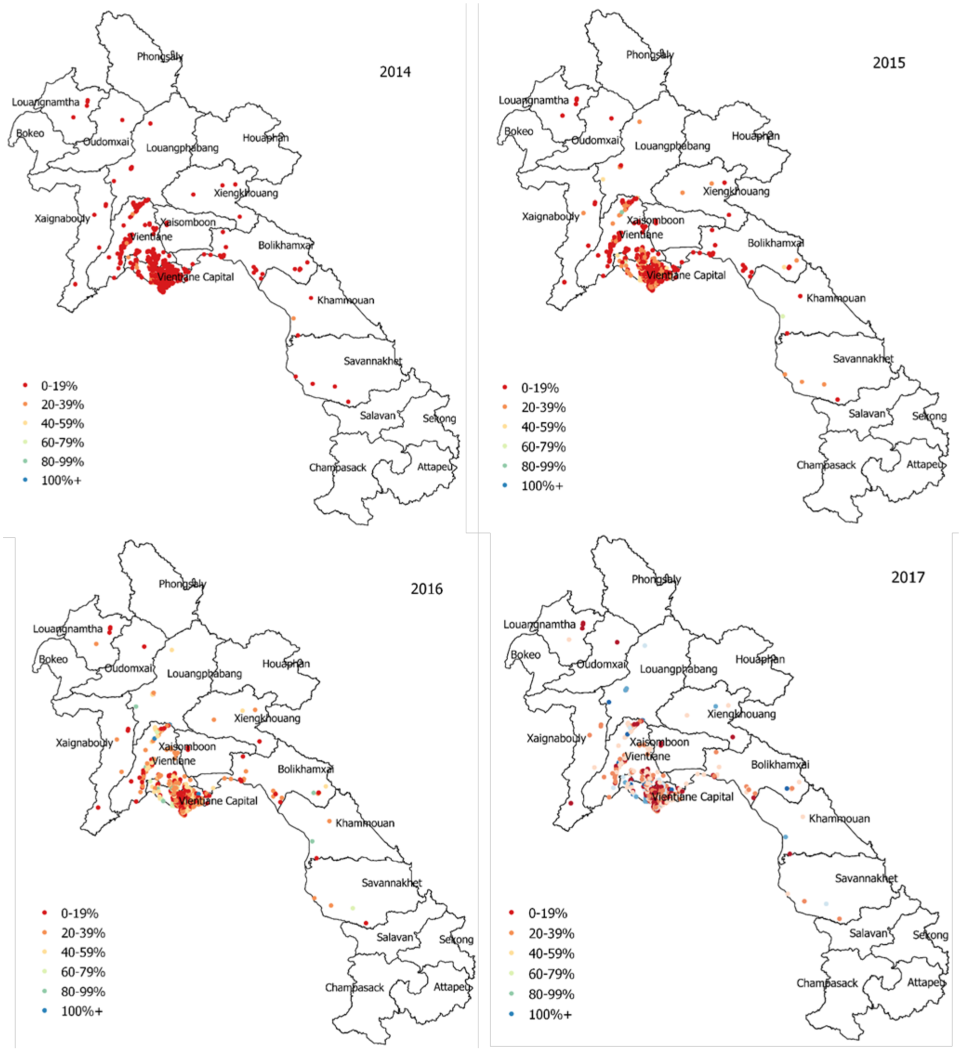

Supplementary figure 3: Map of 13-valent pneumococcal conjugate vaccine (PCV13) coverage among children under five years of age for each case's village of residence, Lao PDR, 2014-2017

Pneumococcal conjugate vaccine coverage

Supplementary table 2: Median* village 13-valent pneumococcal conjugate vaccine (PCV13) coverage calculated among children under five years by year and location, Lao PDR, 2014-2018

	Median PCV13 coverage (IQR)		
	Total (N†=555)	Vientiane Capital (N†=356)	Outside Vientiane Capital (N†=199)
**2014**	4.5 (0.0-10.3)	6.7 (2.9-14.5)	0.7 (0.0-5.0)
**2015**	15.8 (6.1-28.0)	17.3 (8.7-33.4)	11.7 (3.0-22.1)
**2016 (N†=553)**	28.9 (15.9-44.1)	28.4 (15.9-49.5)	30.0 (15.8-40.1)
**2017 (N†=550)**	37.5 (24.1-56.4)	35.2 (23.8-60.3)	40.5 (24.9-51.5)

**2018 (N†=7)**	49.7 (26.1-74.0)	43.3 (26.1-49.7)	70.0 (39.5-82.1)

* Median coverage and IQR among all villages for which we have collected PCV13 coverage data † Number of villages

Supplementary table 3: Median* village 13-valent pneumococcal conjugate vaccine (PCV13) coverage calculated among children 12-23 months of age by year and location, Lao PDR, 2014-2018

	Median PCV13 coverage (IQR)		
	Total (N†=555)	Vientiane Capital (N†=356)	Outside Vientiane Capital (N†=199)
**2014**	0.0 (0.0-11.5)	5.9 (0.0-20.0)	0.0 (0.0-0.0)
**2015**	40.3 (6.7-77.6)	48.8 (22.9-88.0)	22.2 (0.0-50.0)
**2016 (N†=553)**	66.3 (34.5-97.1)	67.6 (35.9-98.7)	59.1 (30.3-90.5)
**2017 (N†=550)**	62.5 (37.8-94.8)	59.7 (28.6-88.9)	71.0 (44.7-102.8)
**2018 (N†=7)**	50.0 (11.5-82.1)	50.0 (37.7-82.1)	44.2 (5.8-95.4)

* Median coverage and IQR among all villages for which we have collected PCV13 coverage data † Number of villages

Supplementary table 4: Crude and adjusted* odds ratios of vaccine-type pneumococcal carriage among children under five, by percent increase in 13-valent pneumococcal conjugate vaccine (PCV13) coverage and individual vaccination status, Vientiane, Lao PDR, December 2013 – June 2018

	Crude		Adjusted*	
	OR (95% CI)	p-value	OR (95% CI)	p-value
**Excluding cases from villages with missing vaccination books (included cases, N=673)**				
**PCV13 coverage**	0.985(0.973-0.997)	0.016	0.993 (0.981-1.004)	0.219
**PCV13 status**	0.520 (0.356-0.759)	0.001	0.583 (0.358-0.949)	0.030
**PCV13 effectiveness (%)†**	48.0 (24.1-64.4)	0.001	41.7 (5.1-64.2)	0.030
**Excluding cases from villages with poor quality vaccination data (included cases, N=67)**				
**PCV13 coverage**	0.984 (0.0.954-1.017)	0.344	0.990 (0.962-1.020)	0.519
**PCV13 status**	0.520 (0.0.356-0.758)	0.001	0.220 (0.036-1.322)	0.098
**PCV13 effectiveness (%)†**	48.0 (24.2-64.4)	0.001	78.0 (-32.2-96.4)	0.098

* adjusted by family income, age, season, number of children under five in the household and kindergarten attendance, †PCV13 effectiveness against VT carriage was one minus the adjusted odds ratio for the association between VT carriage and individual PCV13 status and multiplied by 100

References

1 Hernán MA, Robins JM. Causal Inference: What If. Boca Raton: Chapman & Hall/CRC, 2020.

2 Textor J, van der Zander B, Gilthorpe MS, Liśkiewicz M, Ellison GT. Robust causal inference using

directed acyclic graphs: the R package ‘dagitty’. *Int J Epidemiol* 2016; **45**: 1887–94.
